# Effects of mindfulness-based stress reduction on perioperative outcomes in patients with advanced hepatocellular carcinoma undergoing transarterial chemoembolization

**DOI:** 10.1371/journal.pone.0352434

**Published:** 2026-06-29

**Authors:** Yunyun Zhao, Juanjuan Li, Xiaobei Li, Mao Zhang, Jingxia Qin

**Affiliations:** 1 Interventional Diagnosis and Treatment Center, the First College of Clinical Medical Science, China Three Gorges University, Yichang, China; 2 Interventional Diagnosis and Treatment Center, Yichang Central People’s Hospital, Yichang, China; 3 Department of Interventional Radiology, the First College of Clinical Medical Science, China Three Gorges University, Yichang, China; 4 Department of Interventional Radiology, Yichang Central People’s Hospital, Yichang, China; Hokkaido University: Hokkaido Daigaku, JAPAN

## Abstract

**Objective:**

To evaluate the effects of mindfulness-based stress reduction (MBSR) combined with standard perioperative care on perioperative outcomes in patients with advanced hepatocellular carcinoma (HCC) undergoing transarterial chemoembolization (TACE).

**Methods:**

This retrospective study enrolled patients with advanced HCC who underwent TACE and received either standard perioperative care alone or standard care combined with an MBSR intervention. Post-embolization syndrome (PES) was assessed 72 hours after TACE. Postoperative pain was evaluated using the numerical rating scale (NRS), while anxiety and depression were assessed using the Hospital Anxiety and Depression Scale (HADS). Self-care efficacy was measured using the Strategies Used by People to Promote Health (SUPPH) scale. Univariate and multivariable logistic regression analyses were performed to explore factors associated with PES.

**Results:**

Compared with standard care, the MBSR intervention was associated with significantly lower postoperative NRS pain scores at all assessed time points, reduced use and escalation of analgesic medications, and lower anxiety and depression scores (all *P* < 0.05). Patients in the mindfulness group also demonstrated significantly higher SUPPH scores, indicating better self-care efficacy (*P* < 0.001). In addition, the incidence of PES and postoperative length of hospital stay were significantly lower in the mindfulness group. Logistic regression analysis showed that the mindfulness intervention was independently associated with a reduced risk of PES (OR = 0.55, 95% CI 0.31–0.96).

**Conclusion:**

Mindfulness-based stress reduction combined with standard perioperative care was associated with improved pain control, psychological well-being, self-care efficacy, and reduced perioperative complications in patients with advanced HCC undergoing TACE. This intervention may serve as a feasible supportive approach in perioperative clinical practice.

## Introduction

Hepatocellular carcinoma (HCC) is one of the most common malignancies worldwide and remains a leading cause of cancer-related mortality [1]. Due to its insidious onset, many patients are diagnosed at an intermediate or advanced stage and are no longer candidates for curative treatments. Transarterial chemoembolization (TACE) is an important therapeutic option for patients with advanced HCC and has been widely adopted in clinical practice because of its proven efficacy in delaying tumor progression and improving survival [2].

However, TACE is an invasive procedure and is frequently associated with post-embolization syndrome (PES), which is characterized by pain, fever, nausea, vomiting, and fatigue [3–5]. These symptoms can significantly impair patients’ treatment experience and negatively affect adherence to subsequent therapies. For patients with advanced HCC, repeated TACE procedures are often required, leading to cumulative physical discomfort and psychological burden. Anxiety, depression, and fear related to disease progression and uncertain prognosis are common in this patient population.

Previous studies have shown that negative emotional states not only reduce quality of life but also influence pain perception and stress responses, potentially increasing the need for analgesic medications and delaying postoperative recovery [6–8]. Conventional perioperative nursing care mainly focuses on physiological monitoring, pharmacological pain management, and the prevention of complications, while psychological interventions and strategies addressing patients’ subjective experiences are relatively limited.

Mindfulness-based stress reduction (MBSR) is a structured psychological intervention that emphasizes nonjudgmental awareness and acceptance of present-moment experiences [9–11]. In recent years, MBSR has been increasingly applied in oncology care, with growing evidence suggesting its potential benefits in reducing pain perception, alleviating anxiety and depression, and enhancing self-efficacy and coping capacity [12]. Specifically regarding hepatocellular carcinoma (HCC), preliminary clinical evidence indicates that targeted psychological supports and cognitive behavioral approaches can effectively reduce TACE-related anticipatory anxiety, alleviate post-embolization distress, and enhance compliance [13,14]. However, evidence regarding the application of MBSR in the perioperative management of patients with advanced HCC undergoing TACE remains limited, and its comprehensive effects on pain control, psychological status, and clinical outcomes have not been fully elucidated.

**Fig 1:**
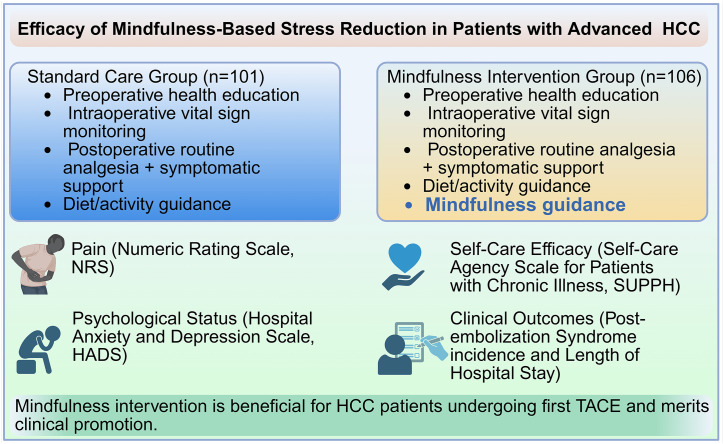
Study flow diagram.

Therefore, this retrospective quasi-experimental study aimed to evaluate the effects of standard care combined with mindfulness-based stress reduction on postoperative pain, psychological status, self-care efficacy, and perioperative clinical outcomes in patients with advanced hepatocellular carcinoma undergoing TACE, with the goal of providing evidence to support the optimization of perioperative nursing care strategies for this population (Fig 1).

## Methods

### Patients

This study was approved by the Institutional Review Board (IRB) of the First College of Clinical Medical Science, China Three Gorges University and was conducted according to the tenets of the 1975 Declaration of Helsinki [15]. Written informed consent was waived for this retrospective study by IRB of the First College of Clinical Medical Science, China Three Gorges University.

**Fig 2:**
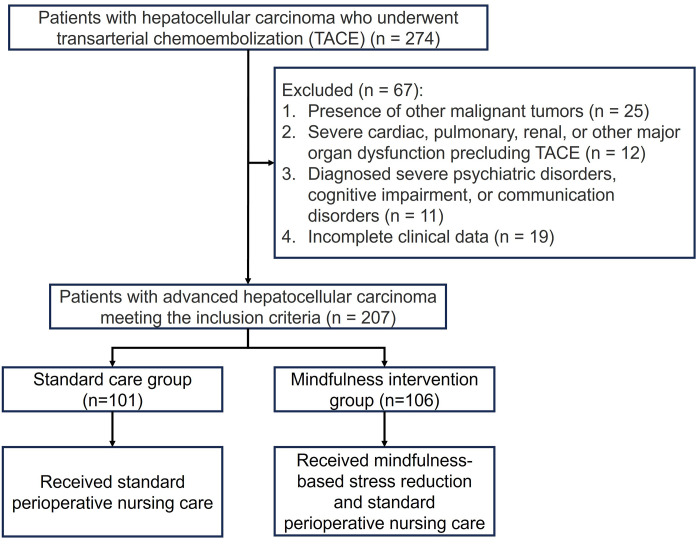
Flow diagram of patient inclusion and exclusion.

This retrospective quasi-experimental study included patients with advanced hepatocellular carcinoma who underwent transarterial chemoembolization in the Interventional Department of our hospital between 01/01/2023 and 31/08/2025. Clinical data were accessed and analyzed for research purposes between 10/09/2025 and 29/12/2025. Patients were assigned to the standard care group or the mindfulness intervention group based on the inpatient ward in which they received perioperative nursing care. It should be noted that this was a non-randomized, natural grouping based on routine hospital triage and bed availability. During the study period, one ward implemented a standardized mindfulness-based stress reduction program in addition to routine perioperative care, whereas the other ward provided routine perioperative care alone (Fig 2).

### Inclusion and exclusion criteria

The inclusion criteria were as follows: (1) a confirmed diagnosis of hepatocellular carcinoma based on imaging or pathological examination; (2) advanced-stage disease defined as Barcelona Clinic Liver Cancer (BCLC) stage B or C; (3) receipt of TACE treatment; (4) Child–Pugh liver function class A or B; (5) Eastern Cooperative Oncology Group (ECOG) performance status score of 0–1; (6) clear consciousness with adequate communication and comprehension abilities to complete relevant assessments; and (7) complete clinical data.

The exclusion criteria were as follows: (1) presence of other malignant tumors; (2) severe dysfunction of the heart, lungs, kidneys, or other major organ systems that precluded tolerance of TACE; (3) diagnosed severe psychiatric disorders, cognitive impairment, or communication disorders; (4) development of severe perioperative complications requiring transfer to the intensive care unit; and (5) incomplete clinical data.

### Perioperative nursing interventions

All patients received routine perioperative nursing care. Preoperatively, responsible nurses provided health education to patients and their family members regarding disease-related knowledge and the TACE procedure, explained perioperative precautions, and helped alleviate anxiety and tension. Intraoperatively, patients’ vital signs were closely monitored. Postoperatively, routine analgesic and symptomatic supportive treatments were administered, with close observation for pain intensity, nausea, vomiting, fever, and other symptoms related to post-embolization syndrome. Patients were also instructed on appropriate diet, moderate physical activity, and post-discharge health management.

In addition to standard care, patients in the mindfulness intervention group received mindfulness-based stress reduction. MBSR was implemented as a standardized nursing program in our department during the study period. The intervention was delivered by specialized oncology nurses who had completed formal MBSR training. To ensure intervention fidelity, the providers were supervised weekly by a certified clinical psychologist. The intervention was strictly standardized, utilizing a unified clinical manual and standardized pre-recorded audio guides for the seated meditation and body scan practices. The MBSR intervention commenced one day before TACE and continued throughout the postoperative hospitalization period. An initial mindfulness session was conducted preoperatively, followed by one session per day postoperatively. Each session lasted approximately 15–30 minutes and was adjusted according to the patient’s tolerance and physical condition.

The intervention included guided seated meditation, during which patients were instructed to focus on their breathing, sounds, and bodily sensations while observing thoughts and emotions in a nonjudgmental manner; systematic body scanning to enhance awareness of tension and discomfort in different body regions and promote relaxation; walking meditation, when physical condition permitted, with attention directed to the sensation of the feet contacting the ground; brief breathing awareness exercises to help patients rapidly recognize their current physical sensations, emotional states, and psychological experiences during episodes of pain or emotional fluctuation; and simple mindfulness-based yoga movements, emphasizing awareness of bodily sensations during movement to promote relaxation and alleviate discomfort under safe conditions. Throughout the intervention, patients were encouraged to participate according to their individual tolerance levels, without emphasizing performance outcomes, and were guided to approach postoperative pain and discomfort with an attitude of awareness and acceptance. Patient adherence to the intervention was routinely monitored and documented in the nursing records. Within the study’s observation window (up to 72 hours postoperatively), the standard protocol comprised 4 scheduled sessions (1 preoperative session and 3 postoperative daily sessions). Given the structured inpatient environment, patient adherence was highly satisfactory, with 95% of the patients in the intervention group attending and completing all 4 scheduled MBSR sessions.

### Outcome measures

Postoperative pain intensity was assessed using the numerical rating scale (NRS) [16] at 0 h, 24 h, 48 h, and 72 h after TACE. Psychological status was evaluated using the Hospital Anxiety and Depression Scale (HADS) [17] at 72 hours after TACE. Self-care efficacy was assessed using the Strategies Used by People to Promote Health (SUPPH) scale, including subdomains of positive attitude, stress coping, and decision participation at 72 hours after TACE. The SUPPH scale has been widely used to assess self-care self-efficacy in nursing research. Clinical outcomes included the use of rescue analgesics, escalation of analgesic regimens, incidence of post-embolization syndrome, and length of postoperative hospital stay. Post-embolization syndrome was defined according to previous studies as the occurrence of one or more of the following symptoms within 72 hours after TACE: abdominal pain requiring analgesic treatment, fever (body temperature ≥38.0 °C), nausea, vomiting, or fatigue, in the absence of other identifiable causes such as infection [5,18,19].

### Statistical Analysis

Statistical analysis was performed using SPSS software. Continuous variables are presented as mean ± standard deviation (SD) or median with interquartile range (IQR), depending on their distribution. Prior to analysis, the normality of continuous data was evaluated using the Shapiro-Wilk test, and the homogeneity of variance was assessed using Levene’s test. Normally distributed data with equal variances were compared between groups using independent-samples t-tests, whereas non-normally distributed data were analyzed using the Mann-Whitney U test. Categorical variables are expressed as frequencies and percentages and were compared using the chi-square test or Fisher’s exact test, as appropriate.

To account for the baseline difference in pain intensity immediately after the procedure, an analysis of covariance (ANCOVA) was further performed for postoperative NRS scores at 24 h, 48 h, and 72 h, using the NRS score at 0 h as a covariate. In addition, because postoperative NRS scores were repeatedly measured within the same patients over time, a linear mixed-effects model was further performed as a sensitivity analysis to account for within-subject correlations. Group, time, and the group × time interaction were included as fixed effects, while patient identity was treated as a random effect. To identify factors associated with the occurrence of post-embolization syndrome, univariate logistic regression analyses were first performed. Subsequently, a multivariable logistic regression model was constructed to evaluate the independent association between the mindfulness intervention and PES after adjustment for clinically relevant covariates. Variables included in the multivariable model were selected based on both clinical relevance and model stability.

Specifically, all variables with a P-value < 0.10 in the univariate analysis were included in the multivariable logistic regression model to ensure robust adjustment for potential confounders while preventing model overfitting. Furthermore, subgroup and sensitivity analyses were performed by stratifying the cohort based on ECOG performance status, Child-Pugh class, BCLC stage, and etiology, with interaction terms explicitly evaluated. Effect sizes for group differences and associations were reported as odds ratios (ORs) with their corresponding 95% confidence intervals (CIs). All statistical analyses were performed using SPSS version 27.0.1 (IBM Corp., Armonk, NY, USA), and a two-sided p value < 0.05 was considered statistically significant.

## Results

### Baseline characteristics

A total of 207 patients with advanced hepatocellular carcinoma undergoing TACE were included in this study, with 101 patients in the standard care group and 106 patients in the mindfulness intervention group. Comparisons of baseline demographic and clinical characteristics between the two groups showed no statistically significant differences in age, sex, ECOG performance status, Child–Pugh class, BCLC stage, etiology, tumor distribution, or tumor size (all P > 0.05), indicating good baseline comparability between the two groups (Table 1).

**Table 1:** Comparison of baseline clinical characteristics between the two groups of patients with advanced hepatocellular carcinoma.

Characteristics	Standard Care Group (n = 101)	Mindfulness Intervention Group (n = 106)	P-value
Age group			0.184
<60 years old	83 (82.2%)	94 (88.7%)	
≥60 years old	18 (17.8%)	12 (11.3%)	
Sex			0.238
Male	83 (82.2%)	80 (75.5%)	
Female	18 (17.8%)	26 (24.5%)	
ECOG PS			0.174
0	30 (29.7%)	41 (38.7%)	
1	71 (70.3%)	65 (61.3%)	
Child-Pugh class			0.521
A	80 (79.2%)	80 (75.5%)	
B	21 (20.8%)	26 (24.5%)	
BCLC stage			0.149
A	0 (0%)	1 (0.9%)	
B	26 (25.7%)	17 (16%)	
C	75 (74.3%)	88 (83%)	
Etiology			0.208
Hepatitis B	84 (83.2%)	83 (78.3%)	
Others	17 (16.8%)	26 (24.5%)	
Tumor distribution			0.476
Single	26 (25.7%)	32 (30.2%)	
Multiple	75 (74.3%)	74 (69.8%)	
Tumor size			0.322
<10 cm	43 (42.6%)	38 (35.8%)	
≥10 cm	58 (57.4%)	68 (64.2%)	

TACE, transarterial chemoembolization; ECOG PS, Eastern Cooperative Oncology Group performance status; BCLC, Barcelona Clinic Liver Cancer staging system. Categorical variables are presented as number (%), and comparisons between groups were performed using the chi-square test.

### Comparison of postoperative pain scores

**Fig 3:**
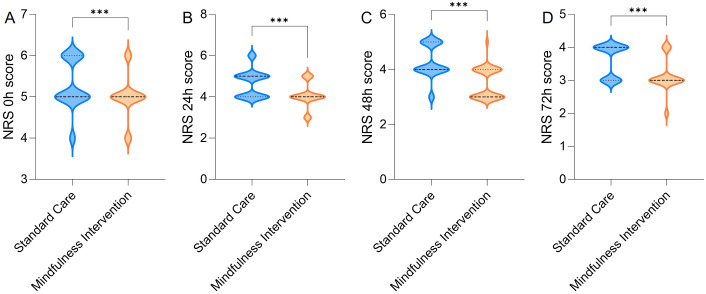
Comparison of Postoperative Pain Scores (NRS) at Different Time Points Between the Two Groups. NRS, numerical rating scale, ranging from 0 to 10, with higher scores indicating more severe pain. Comparisons between groups were conducted using independent-samples t tests. *P < 0.05, **P < 0.01, ***P < 0.001.

Postoperative pain intensity assessed by the numerical rating scale at 0 h, 24 h, 48 h, and 72 h after TACE is shown in Table 2 and Fig 3. The mindfulness intervention group demonstrated significantly lower NRS scores at all postoperative time points compared with the standard care group (all P < 0.001). Furthermore, after adjusting for the initial baseline difference at 0 h using ANCOVA, the mindfulness group maintained significantly lower pain scores at 24 h, 48 h, and 72 h (all adjusted P < 0.001), confirming the independent effect of MBSR on longitudinal pain control. In addition, longitudinal sensitivity analysis using a linear mixed-effects model demonstrated significant effects of group, time, and group × time interaction on postoperative NRS scores (all P < 0.001), confirming the robustness of the observed longitudinal pain differences between groups (S1 Table). These results indicate that the addition of mindfulness-based stress reduction was associated with improved postoperative pain control.

**Table 2:** Comparison of postoperative numerical rating scale and hospital anxiety and depression scale and strategies used by people to promote health scale between the two groups.

	Standard Care Group (n = 101)	Mindfulness Intervention Group (n = 106)	P-value	Adjusted P-value
NRS 0h	5.28 ± 0.60	5.00 ± 0.52	<0.001	–
NRS 24h	4.65 ± 0.66	4.08 ± 0.58	<0.001	<0.001
NRS 48h	4.28 ± 0.60	3.50 ± 0.56	<0.001	<0.001
NRS 72h	3.62 ± 0.49	3.12 ± 0.49	<0.001	<0.001
HADS-Anxiety	7.67 ± 0.99	6.17 ± 1.24	<0.001	–
HADS-Depression	7.08 ± 1.12	5.46 ± 0.98	<0.001	–
SUPPH	86.22 ± 4.14	93.00 ± 5.67	<0.001	–
SUPPH-positive attitude	28.10 ± 1.73	31.29 ± 2.47	<0.001	–
SUPPH-stress coping	26.46 ± 1.68	29.29 ± 2.44	<0.001	–
SUPPH-decision participation	30.97 ± 1.87	32.83 ± 2.66	<0.001	–

NRS, numerical rating scale, ranging from 0 to 10, with higher scores indicating more severe pain. HADS, Hospital Anxiety and Depression Scale, including anxiety and depression subscales; SUPPH, Strategies Used by People to Promote Health scale, including subdomains of positive attitude, stress coping, and decision participation. Comparisons between groups were conducted using independent-samples t tests. Adjusted P-values were calculated using analysis of covariance (ANCOVA) for longitudinal NRS scores, with the baseline NRS score at 0 h included as a covariate.

### Comparison of psychological status and self-care efficacy

**Fig 4:**
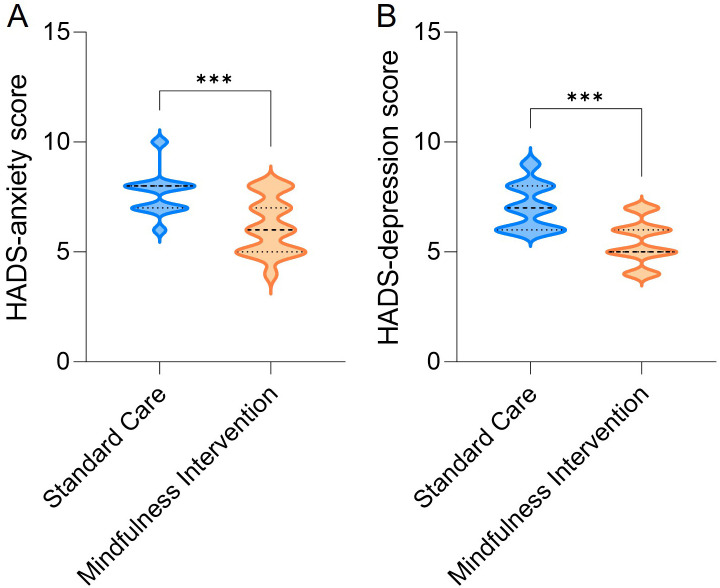
Comparison of hospital anxiety and depression scale between the two groups. HADS, Hospital Anxiety and Depression Scale, including anxiety and depression subscales; SUPPH, Strategies Used by People to Promote Health scale, including subdomains of positive attitude, stress coping, and decision participation. Comparisons between groups were conducted using independent-samples t tests. *P < 0.05, **P < 0.01, ***P < 0.001.

**Fig 5:**
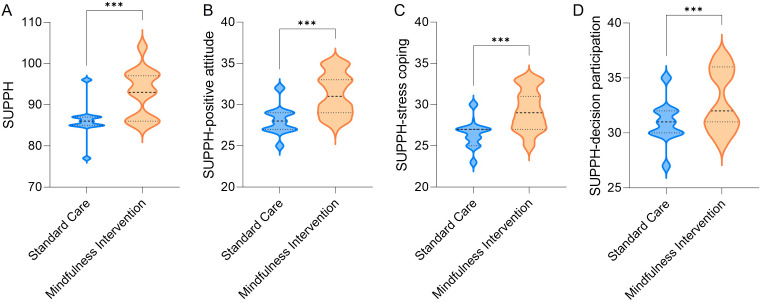
Comparison of strategies used by people to promote health scale between the two groups. SUPPH, Strategies Used by People to Promote Health scale, including subdomains of positive attitude, stress coping, and decision participation. Comparisons between groups were conducted using independent-samples t tests. *P < 0.05, **P < 0.01, ***P < 0.001.

Psychological status and self-care efficacy outcomes are summarized in Table 2. The mindfulness intervention group had significantly lower HADS anxiety and depression scores than the standard care group (both P < 0.001) (Fig 4). Furthermore, total SUPPH scores and scores across all SUPPH subdimensions were significantly higher in the mindfulness group compared with the standard care group (all P < 0.001), indicating improved self-care efficacy (Fig 5).

### Comparison of analgesic use

The proportion of patients requiring rescue analgesics was significantly lower in the mindfulness intervention group than in the standard care group (17.9% vs. 32.7%, P = 0.014) (Table 3). In addition, the rate of escalation of analgesic regimens was also significantly reduced in the mindfulness group compared with the standard care group (11.3% vs. 25.7%, P = 0.007).

**Table 3:** Comparison of postoperative analgesic use and clinical outcomes between the two groups.

	Standard Care Group (n = 101)	Mindfulness Intervention Group (n = 106)	P-value
Rescue analgesics	33 (32.7%)	19 (17.9%)	0.014
Escalation of analgesic regimen	26 (25.7%)	12 (11.3%)	0.007
Incidence rate of post-embolization syndrome	49 (48.5%)	37 (34.9%)	0.047
Length of postoperative hospital stay	6.60 ± 0.81	5.78 ± 0.91	<0.001

Post-embolization syndrome refers to a cluster of symptoms occurring after TACE, including pain, fever, nausea, and vomiting. Length of postoperative hospital stay is expressed in days (d). Categorical variables were analyzed using the chi-square test, and continuous variables were analyzed using independent-samples t tests.

### Comparison of clinical outcomes

The incidence of post-embolization syndrome was significantly lower in the mindfulness intervention group than in the standard care group (34.9% vs. 48.5%, P = 0.047) (Table 3). The component-level breakdown of symptoms among patients with PES is detailed in S2 Table, with abdominal pain remaining the predominant manifestation in both groups (85.7% vs. 83.8%). In addition, the length of postoperative hospital stay was significantly shorter in the mindfulness group compared with the standard care group (P < 0.001).

Univariate logistic regression analysis showed that the mindfulness intervention was significantly associated with a lower risk of post-embolization syndrome (OR = 0.57, 95% CI 0.33–0.99, p = 0.048). Tumor distribution showed a borderline association with PES, whereas age, sex, ECOG performance status, tumor size, Child–Pugh class, and hepatitis B virus infection were not significantly associated with PES in univariate analyses. In multivariable logistic regression analysis adjusting for clinically relevant covariates, the mindfulness intervention remained independently associated with a reduced risk of PES (adjusted OR = 0.55, 95% CI 0.31–0.96, p = 0.037) (Table 4). Subgroup analyses further demonstrated that the protective effect of MBSR against PES was consistent across strata defined by ECOG performance status, BCLC stage, and etiology (all P for interaction > 0.05) (S1 Fig). Notably, a significant interaction was observed for Child-Pugh class (P for interaction = 0.008), indicating that the MBSR intervention conferred a particularly profound protective effect in patients with Child-Pugh B liver function (OR = 0.10, 95% CI 0.02–0.43, P = 0.002).

**Table 4:** Univariate and multivariable logistic regression analyses of factors associated with post-embolization syndrome.

Characteristics	Univariate			Multivariate		
OR	95%CI	P-value	OR	95%CI	P-value
Group (mindfulness intervention)	0.569	0.326-0.995	0.048	0.547	0.311-0.963	0.037
Age group (≥60 vs. <60)	1.089	0.499-2.381	0.830			
Gender (Female vs. Male)	0.759	0.382-1.511	0.433			
ECOG PS (1 vs. 0)	0.957	0.535-1.712	0.881			
Tumor size (≥10 cm vs. <10 cm)	1.147	0.659-1.996	0.628			
Tumor distribution (multiple vs. single)	0.562	0.305-1.037	0.065	0.536	0.288-0.999	0.052
Child-Pugh class (B vs. A)	0.588	0.295-1.169	0.130			
HBV (yes vs. no)	1.191	0.594-2.387	0.622			

PES, post-embolization syndrome; OR, odds ratio; CI, confidence interval; ECOG PS, Eastern Cooperative Oncology Group performance status; HBV, hepatitis B virus.

## Discussion

Transarterial chemoembolization is an important therapeutic modality for patients with advanced hepatocellular carcinoma [20]. However, postoperative pain and post-embolization syndrome remain common clinical challenges [21]. In addition to ischemic injury and inflammatory responses induced by tumor necrosis, psychological factors play a critical role in shaping patients’ pain perception and postoperative recovery [22]. Identifying effective nursing interventions to improve perioperative experiences and outcomes has therefore become an important focus in clinical practice.

While mindfulness-based interventions have proven effective in alleviating chronic symptom burden across various malignancies [23,24], evidence regarding their application in acute, procedure-related settings remains scarce. By significantly reducing PES and acute postoperative pain, our study expands the utility of MBSR from a long-term psychosocial support tool to an effective non-pharmacological adjunct for acute perioperative care. The present study demonstrated that, compared with standard care alone, the addition of mindfulness-based stress reduction was associated with significantly lower postoperative pain scores at multiple time points following TACE, as well as reduced use of rescue analgesics and lower rates of escalation of analgesic regimens. These findings suggest that mindfulness-based interventions may contribute to improved pain management in patients with advanced hepatocellular carcinoma undergoing TACE. Previous studies have indicated that pain perception is influenced not only by physiological injury but also by cognitive and emotional processes. By encouraging nonjudgmental awareness and acceptance of present-moment experiences, MBSR may help attenuate the amplification of pain associated with anxiety and emotional distress [10].

In terms of psychological outcomes, patients in the mindfulness intervention group exhibited significantly lower levels of anxiety and depression compared with those receiving standard care. Patients with advanced hepatocellular carcinoma often experience persistent psychological stress related to disease progression, repeated interventional treatments, and uncertainty regarding prognosis. Mindfulness-based interventions emphasize awareness and acceptance of emotional experiences, which may reduce excessive rumination and anticipatory anxiety related to future outcomes. The present findings are consistent with previous reports suggesting that MBSR may be beneficial for alleviating anxiety and depressive symptoms in oncology populations.

Self-care efficacy is a key psychological factor influencing patients’ engagement in symptom management and treatment-related decision-making [25,26]. In this study, SUPPH total scores and subscale scores were significantly higher in the mindfulness intervention group, indicating enhanced self-care efficacy. Through mindfulness practice, patients may develop greater awareness of bodily sensations and emotional responses, thereby potentially enhancing their capability to cope with symptoms and actively participate in their care. This improvement in self-efficacy may partially explain the observed benefits in pain control and psychological well-being.

Regarding clinical outcomes, the incidence of post-embolization syndrome was lower and the length of postoperative hospital stay was shorter in the mindfulness intervention group. Post-embolization syndrome is closely associated with pain severity, inflammatory responses, and overall discomfort. Better pain management and psychological regulation may be associated with an alleviated symptom burden and facilitated postoperative recovery, which in turn could correlate with a shorter hospitalization duration [27,28].

Beyond psychological pathways, the benefits of MBSR may also involve neuroendocrine and inflammatory mechanisms. TACE-induced tumor necrosis triggers systemic inflammation. Psychological stress can exacerbate this trauma by overactivating the hypothalamic-pituitary-adrenal (HPA) axis, amplifying pro-inflammatory cytokine release. Evidence suggests mindfulness practices can buffer this stress response, downregulate HPA hyperactivity, and subsequently attenuate the inflammatory cascade [29,30]. This psychoneuroimmunological modulation provides a plausible biological rationale for the reduced PES incidence and improved pain control observed in our study.

Interestingly, tumor size and Child-Pugh class were not significantly associated with PES in our cohort. This lack of association likely reflects real-world clinical adaptations. To mitigate hepatic failure risks, patients with massive tumors (≥ 10 cm) or compromised liver function (Child-Pugh B) typically receive more conservative or staged embolization, alongside intensified prophylactic perioperative care. These protective procedural modifications likely offset their inherent biological vulnerabilities, resulting in comparable PES incidences across these subgroups.

Despite the observed benefits, implementing MBSR in routine clinical practice faces practical challenges. First, time constraints in busy interventional wards can be mitigated by utilizing pre-recorded audio guides and standardizing brief sessions (15–30 minutes), as demonstrated in our study. Second, regarding staffing requirements, training a core group of oncology nurses with periodic psychological supervision offers a more scalable and pragmatic solution than relying solely on full-time psychologists. Finally, to optimize patient acceptance, particularly among those experiencing severe postoperative fatigue or skepticism toward psychological interventions, MBSR should be framed as a routine component of supportive pain management, with flexible scheduling tailored to individual tolerance.

Several limitations of this study should be acknowledged. First, the retrospective design and the non-randomized, natural grouping method based on inpatient wards may introduce selection bias. Although baseline characteristics were comparable between the two groups, unmeasured confounders associated with ward-specific practices cannot be entirely ruled out, and causal relationships cannot be definitively established. Second, an inherent selection bias may also arise from variations in patient motivation, as individuals with higher adherence or compliance might have been disproportionately represented in the mindfulness group. Third, this was a single-center study with a relatively limited sample size, which may restrict the generalizability of the findings. Fourth, our observation window was restricted to the acute perioperative period (up to 72 hours), precluding the assessment of long-term psychological well-being and oncological outcomes. Fifth, we failed to formally evaluate patient satisfaction with the MBSR intervention, which remains an important metric for clinical refinement. In addition, the effectiveness of mindfulness-based interventions may be influenced by individual differences in patients’ physical condition, psychological readiness, and engagement with the intervention. Future prospective, multicenter randomized controlled trials are warranted to further clarify the long-term effects and underlying mechanisms of MBSR in patients with advanced hepatocellular carcinoma undergoing TACE.

In conclusion, the findings of this study suggest that integrating mindfulness-based stress reduction into standard perioperative nursing care is positively associated with more favorable pain trajectories, better psychological well-being, higher self-care efficacy, and a lower incidence of perioperative complications in patients with advanced hepatocellular carcinoma undergoing TACE. Given its feasibility and safety, this intervention may represent a valuable adjunct to routine nursing care in interventional oncology settings.

## Supporting information

S1 Fig Subgroup analyses of the effect of MBSR on PES incidence. (DOCX)

S1 Table Linear mixed-effects model analysis of longitudinal postoperative NRS scores. (DOCX)

S2 Table Component-level breakdown of symptoms among patients who developed post-embolization syndrome. (DOCX)

S1 File The minimal dataset. (XLSX)
