# Supplementary material for: Effects of mindfulness-based stress reduction on perioperative outcomes in patients with advanced hepatocellular carcinoma undergoing transarterial chemoembolization
Source: PLoS One. 2026 Jun 29;21(6):e0352434. doi: 10.1371/journal.pone.0352434 (PMC13313351; doi:10.1371/journal.pone.0352434)
Supplement: S1 Fig — (DOCX) [file pone.0352434.s001.docx]

**S1 Fig. Subgroup analyses of the effect of MBSR on PES incidence.**


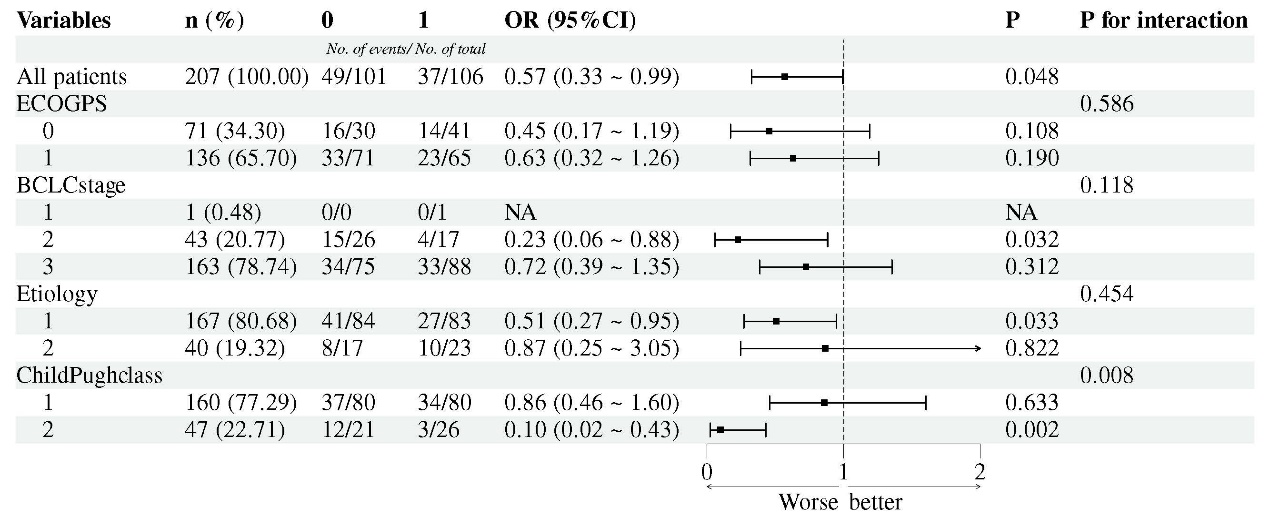


The forest plot displays the odds ratios (ORs) and 95% confidence intervals (CIs). *Abbreviations:* MBSR, mindfulness-based stress reduction; PES, post-embolization syndrome; ECOG PS, Eastern Cooperative Oncology Group performance status; BCLC, Barcelona Clinic Liver Cancer.
