# Supplementary material for: Effects of mindfulness-based stress reduction on perioperative outcomes in patients with advanced hepatocellular carcinoma undergoing transarterial chemoembolization
Source: PLoS One. 2026 Jun 29;21(6):e0352434. doi: 10.1371/journal.pone.0352434 (PMC13313351; doi:10.1371/journal.pone.0352434)
Supplement: S1 Table — (DOCX) [file pone.0352434.s002.docx]

**S1 Table. Linear mixed-effects model analysis of longitudinal postoperative NRS scores**

| **Fixed effects** | **Estimate** | **SE** | **z value** | **P-value** |
| --- | --- | --- | --- | --- |
| Intercept | 5.277 | 0.056 | 94.123 | <0.001 |
| Time (24 h vs. 0 h) | -0.624 | 0.042 | -14.725 | <0.001 |
| Time (48 h vs. 0 h) | -1 | 0.042 | -23.606 | <0.001 |
| Time (72 h vs. 0 h) | -1.653 | 0.042 | -39.032 | <0.001 |
| Group (Mindfulness vs. Standard care) | -0.277 | 0.078 | -3.538 | <0.001 |
| Group × Time (24 h) | -0.301 | 0.059 | -5.081 | <0.001 |
| Group × Time (48 h) | -0.5 | 0.059 | -8.446 | <0.001 |
| Group × Time (72 h) | -0.224 | 0.059 | -3.782 | <0.001 |

Linear mixed-effects model including fixed effects for group, time, and group × time interaction, with patient identity treated as a random effect to account for within-subject correlations. Reference categories were standard care group and 0 h postoperative NRS assessment.
