# Supplementary material for: Effects of mindfulness-based stress reduction on perioperative outcomes in patients with advanced hepatocellular carcinoma undergoing transarterial chemoembolization
Source: PLoS One. 2026 Jun 29;21(6):e0352434. doi: 10.1371/journal.pone.0352434 (PMC13313351; doi:10.1371/journal.pone.0352434)
Supplement: S2 Table — (DOCX) [file pone.0352434.s003.docx]

**S2 Table. Component-level breakdown of symptoms among patients who developed post-embolization syndrome.**

| Symptoms | Standard Care Group (n=49) | Mindfulness Intervention Group (n=37) |
| --- | --- | --- |
| Abdominal pain | 42 (85.7%) | 31 (83.8%) |
| Fever | 38 (77.6%) | 28 (75.7%) |
| Nausea | 29 (59.2%) | 21 (56.8%) |
| Vomiting | 15 (30.6%) | 12 (32.4%) |
